# Exosomes as A Next-Generation Diagnostic and Therapeutic Tool in Prostate Cancer

**DOI:** 10.3390/ijms221810131

**Published:** 2021-09-20

**Authors:** Simita Gaglani, Edgar Gonzalez-Kozlova, Dara J. Lundon, Ashutosh K. Tewari, Navneet Dogra, Natasha Kyprianou

**Affiliations:** 1 Department of Urology, Icahn School of Medicine at Mount Sinai, 1425 Madison Avenue, New York, NY 10029, USA; Simita.Gaglani01@utrgv.edu (S.G.); Dara.Lundon@mountsinai.org (D.J.L.); Ash.Tewari@mountsinai.org (A.K.T.); 2 Department of Pathology and Molecular and Cell Based Medicine, Icahn School of Medicine at Mount Sinai, New York, NY 10029, USA; Edgar.Gonzalez-Kozlova@mssm.edu; 3 Department of Genetics and Genomic Sciences, Icahn School of Medicine at Mount Sinai, 1468 Madison Avenue, New York, NY 10029, USA; 4 Tisch Cancer Institute, Icahn School of Medicine at Mount Sinai, New York, NY 10029, USA; 5 Department of Oncological Sciences, Icahn School of Medicine at Mount Sinai, New York, NY 10029, USA

**Keywords:** prostate cancer, PSA, extracellular vesicles, exosomes, biomarkers, liquid biopsy, therapeutic resistance

## Abstract

Extracellular vesicles (EVs) have brought great momentum to the non-invasive liquid biopsy procedure for the detection, characterization, and monitoring of cancer. Despite the common use of PSA (prostate-specific antigen) as a biomarker for prostate cancer, there is an unmet need for a more specific diagnostic tool to detect tumor progression and recurrence. Exosomes, which are EVs that are released from all cells, play a large role in physiology and pathology, including cancer. They are involved in intercellular communication, immune function, and they are present in every bodily fluid studied—making them an excellent window into how cells are operating. With liquid biopsy, EVs can be isolated and analyzed, enabling an insight into a potential therapeutic value, serving as a vehicle for drugs or nucleic acids that have anti-neoplastic effects. The current application of advanced technology also points to higher-sensitivity detection methods that are minimally invasive. In this review, we discuss the current understanding of the significance of exosomes in prostate cancer and the potential diagnostic value of these EVs in disease progression.

## 1. Introduction

Prostate cancer is the second most commonly diagnosed cancer in men globally, preceded only by lung cancer [1,2,3]. With an incidence of over 1.4 million in 2020, and being the fifth greatest cause of cancer-related mortality, the need for more effective diagnosis and treatment for prostate cancer is clear [2]. According to the American Cancer Society, one in eight men in the United States develop invasive prostate cancer throughout their lives, and when diagnosed with distant metastases, the five-year survival is 30% [4]. The recurrence rates after radical treatment are between 20 and 30% [5]. Approximately 10–20% of people diagnosed with prostate cancer develop castration-resistant prostate cancer (CRPC) within five years, and over 84% of those patients develop metastases [6]. Metastatic CRPC (mCRPC) has a very poor prognosis, with a mean survival time of 14 months after diagnosis [7]. There are concerns regarding the safety and diagnostic yield of prostate biopsy. Although performed very regularly, prostate biopsies carry the risk of severe infection, due to the needle’s ability to introduce rectal commensal or other bacteria to the sterile prostate. This, in addition to the risks of urinary retention, hematuria, and hematochezia in some, demonstrates the need for a less invasive, but equally efficacious, diagnostic tool [8]. 

Serum biomarkers have been used for decades, as a measure of the presence of prostate cancer. From the 1980s through to the 1990s, prostate-specific antigen (PSA) became the biomarker of choice to monitor cancer progression, recurrence, and detect its presence when combined with abnormal clinical findings [9]. While PSA screening in asymptomatic patients has led to a decreased number of men being found with metastatic disease, it has concomitantly led to the gross overdiagnosis and overtreatment of those with indolent disease [9,10,11]. PSA also lacks the ability to differentiate cancer from other causes, such as benign prostatic hyperplasia (BPH), trauma, and prostatitis [9]. Data from the prostate cancer prevention trial (PCPT) revealed that 14.9% of prostate tumors in men with PSA levels lower than 4.0 ng/mL had Gleason scores of seven or higher [12]. Additionally, while PSA monitoring after prostate cancer treatment has shown to benefit identifying recurrent disease, it serves a much more limited purpose in locating that recurrent disease [5]. It has even less utility for patients who were treated with focal therapy, due to higher amounts of healthy prostatic tissue producing PSA remaining after treatment [13]. Moreover, PSA does not have the capability to predict the response to the treatment of CRPC with hormone therapy or chemotherapy [14].

Other prostate cancer biomarkers have been explored more recently, but with limited use. Prostate cancer antigen 3 (PCA3), for example, is a long noncoding RNA (lncRNA) that is detectable in urine, which has been shown to be elevated in more than 90% of patients with known prostate cancer [9,15]. SelectMDx is another urine-based test and measures the RNA levels of DLX1 and HOXC6 genes; it uses an algorithm including a digital rectal exam (DRE), total PSA, PSA density, age, and family history to provide the risk of high-grade prostate cancer. In their development studies, they demonstrated that 42% of biopsies could be avoided, while missing 2% of high-grade prostate cancers [16]. TMPRSS2:ERG gene fusions are also specific to prostate cancer and can be detected in urine, but are absent in about 50% of patients with prostate cancer [9,17]. The 4Kscore uses clinical variables and serum biomarkers (total PSA, free PSA, intact PSA, and human kallikrein 2) to predict the risk of high-grade prostate cancer on biopsy, and categorizes the risk of prostate cancer metastases and mortality. It has been shown to have a negative predictive value of 95% for the detection of high-grade prostate cancer [18,19]. Circulating tumor cells (CTCs), released from primary tumor sites and metastases into circulation, have been studied as a diagnostic tool for prostate cancer, but challenges in early detection limit their clinical value [9,20]. Extracellular vesicles (EVs) have brought new promise to the diagnosis and treatment of prostate cancer. Liquid biopsies present the opportunity for personalized molecular screening of prostate cancer patients, without significant risks or costs associated with obtaining samples, which makes them an ideal candidate for diagnostics.

## 2. Discussion

### 2.1. Exosomes

EVs have long been known to be released by all cells [21,22,23], with two significant categories of these vesicles, separated by size and origin [24,25,26]. Microvesicles range from 50 nm to 1 μm in size and exit cells via budding outward from the plasma membrane, while exosomes are smaller, from 40 to 160 nm in size, and have endosomal origin [26]. Exosomes functionally enable the cells to expel waste and little else, and also contribute to the immune response, pregnancy, intercellular communication, neurodegenerative disease, cardiovascular disease, and cancer, which has been further explored [21,27].

A better understanding of the role that EVs and their biogenesis play in physiology and normal homeostasis will enable new insights into their functional contribution to cancer development and progression [3]. The biogenesis of exosomes begins with the invagination of a cell’s plasma membrane, to create an intracellular vesicle with reverse membrane orientation [26]. This structure, called an early sorting endosome (ESE), may then fuse with other already present ESEs from cellular organelles such as the endoplasmic reticulum and Golgi network, to eventually form a late sorting endosome (LSE) [28]. The second invagination of the LSE creates smaller intraluminal vesicles (ILVs), which will eventually become exosomes, stored within a multivesicular body (MVB). This process is mediated by endosomal sorting complexes required for transport (ESCRT) proteins, whereby four complexes of 30 ESCRT proteins along the outside of the LSE allow for the ILVs containing ubiquitinated cargo to form within the MVB. ESCRT-independent processes for ILV formation, involving tetraspanins and lipid membrane ceramide, have been observed as well [29,30,31,32,33]. From here, MVBs may either be degraded via lysosomes or transported to the cell membrane for exocytosis, with the assistance of MVB docking proteins, leaving the exosomes and their contents to circulate and be eliminated or taken up by other cells [21,26,29]. Exosome secretion from the parent cell involves the Rab GTPases 27a and 27b, as demonstrated by Ostrowski et al. [34]

**Figure 1:**
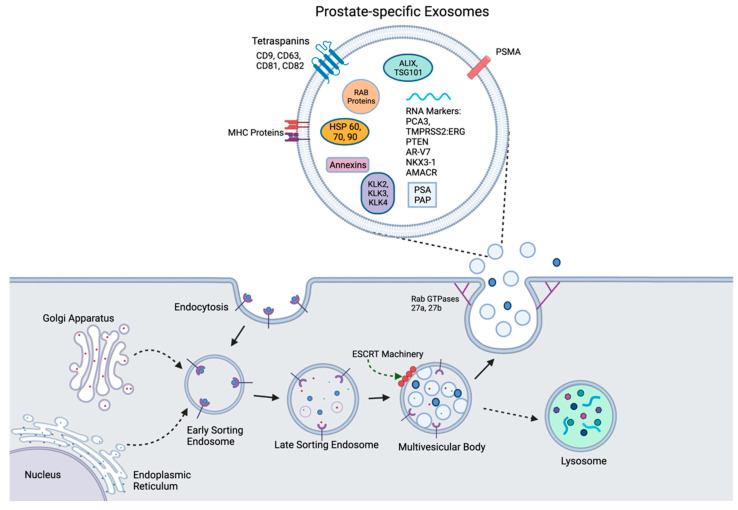
Biogenesis of prostatic exosomes. Endocytosis via cell membrane invagination creates an early sorting endosome (ESE), which combines with other ESEs from the Golgi apparatus and the endoplasmic reticulum to create a late sorting endosome (LSE). Via ESCRT-dependent or ESCRT-independent mechanisms, further invagination creates ILVs within a multivesicular body (MVB). The MVB may either be shuttled to a lysosome for degradation or to the cell surface with the help of MVB docking proteins, such as Rab GTPases 27a and b, to expel its contents, including exosomes, out of the cell. Prostate-specific exosomes (top) contain nonspecific exosome biomarkers, such as heat shock proteins and tetraspanins, while also including kallikreins, PSMA, PCA3, and TMPRSS2:ERG, which are specific to exosomes of prostatic origin.

The contents within exosomes include a wide range of components—several proteins, mRNA, and other noncoding RNA (ncRNA), DNA, and lipids [26,30,35,36,37]. Some proteins are similarly present across exosomes of variable origin. Examples of these are the tetraspanin proteins CD9, CD63, CD81, and CD82; Rab GTPases and ESCRT complex proteins; and the heat shock proteins Hsp 60, Hsp 70, and Hsp 90 [38]. Other components are specific to the cell of origin, as shown in Figure 1. The uptake of exosomes into cells can happen in several potential ways, including phagocytosis, macropinocytosis, direct fusion with the plasma membrane of the recipient cell, interaction with a receptor, or via clathrin- or caveolin-mediated endocytosis [30,39]. Aside from exosomes, EVs such as apoptotic vesicles, microvesicles, and oncovesicles are also released from cells [24,25]. All of these other EVs carry molecules that are specific to their cell of origin, but exosomes have been shown to hold proteins and nucleic acid without concomitantly carrying cellular debris [24,36].

### 2.2. The Functional Contribution of Exosomes to Physiological Homeostasis

#### 2.2.1. Cell Communication and Signaling

Exosomes have been shown to be involved in intercellular communication and signaling [40]. They contain both functional mRNA and ncRNA, which, when transferred from the parent cell to the recipient cell, may induce changes in gene expression, protein translation, and cellular function [36]. For example, Valadi et al. used mast cell exosomes from mice to demonstrate that they can be used as a vessel to transfer novel functions to recipient human mast cells [36]. Significantly enough, the microenvironment dynamics, including heat shock, hypothermia, hypoxia, and oxidative stress, can modify the exosome composition, as does the activation of cytokine-mediated intercellular signaling pathways [3,41]. This suggests that exosomes play a role in cell-to-cell communication and interoperability.

#### 2.2.2. Immunity

Exosome contribution to immune function has been observed as well. Antigen-presenting cells (APCs) secrete exosomes, which contain MHC-II complexed with the antigen, as well as costimulatory signals. These exosomes are sufficient to activate T cells, although the activation is not as effective as it would be if the APC itself had presented the antigen [26]. Even so, the absence of exosomes throughout this process results in greatly diminished antigen presentation [42]. When comparing dendritic cells and their exosomes’ abilities to induce the T cell response, Segura et al. demonstrated that mature dendritic cells contain more MHC-II and ICAM-1, and therefore can elicit a greater response than immature dendritic cells [43]. The exosome shock proteins Hsp60 and Hsp70 have been shown to contribute to dendritic cell antigen presentation [44].

#### 2.2.3. Embryonic Development

Exosomes can also have immunosuppressive effects, as can be observed in pregnancy [45]. Via exosomes released by embryonic cells, maternal immune cells that uptake these exosomes can modulate this immunosuppression that allows for successful pregnancy and fetal development [46]. Placental exosomes contain the Fas ligand (FasL), which is responsible for preventing maternal cytotoxic T cells and natural killer cells from acting on the fetus [41]. Angiogenesis is another process that is stimulated by placental exosomes under the hypoxic conditions of early pregnancy [45]. The additional roles of exosomes in embryonic development include sperm maturation, egg fertilization, polyspermy prevention, and implantation of the embryo [45]. The cross-talk between fetal and maternal cells via exosomes is critical to the physiological prenatal development.

### 2.3. Extracellular Vesicles in Cancer

Under physiological conditions, exosomes contribute to normal cellular function; their role under pathological conditions, however, and, notably, cancer is much different (Table 1). Exosomes are dynamically involved in inducing and advancing tumor development, impacting the landscape of the tumor microenvironment, and immune system activation via effects on vascularity and cell polarity, as well as phenotypic configuration in the context of the tumor microenvironment (epithelial–mesenchymal transition—EMT; and interconversion to mesenchymal–epithelial transition—MET) in several human malignancies [32]. 

In glioma, exosomes are used as a vehicle to transport EGFRvIII, a mutant epidermal growth factor, to cells that do not have it—inducing the expression of anti-apoptotic genes and increasing the anchorage-independent growth capacity [47]. In pancreatic cancer, Stefanius et al. found that cancer cell exosomes have the ability to instigate malignant cell transformation [48]. Colon cancer cells expressing mutant KRAS have been shown to enhance the invasiveness of wild-type KRAS cells via exosomes released by the mutant cells [49]. Exosomes have been shown to aid in tumor growth and progression in malignant disease, due to their ability to suppress immunity. Exosomes can also navigate the therapeutic response of human cancers. Drugs such as doxorubicin and cisplatin have even been shown to be exported from cells via exosomes, demonstrating their contribution to therapeutic resistance [42]. Docetaxel-resistant prostate and breast cancer-derived exosomes have also been shown to confer resistance to sensitive cells [50,51]. 

Post-translational modifications on EVs also have implications in pathogenesis and tumor development, in addition to the physiologic effects that ubiquitination has on their creation. Exosomes that are rich in Wnt5b, a protein that is post-translationally glycan- and lipid-modified, have been associated with head and neck squamous cell carcinomas, invasive breast cancer, and lung and pancreatic cancers [55]. EVs from an ovarian carcinoma line were found to be enriched with certain mannose and sialic acid residues [56]. Src-phosphorylation plays a role in the angiogenesis of myeloid leukemia that is stimulated by exosomes, and this phosphorylation can be therapeutically targeted [57].

The functional contribution of exosomes to malignant growth implicates their value as a less invasive liquid biopsy method for detecting advanced prostate cancer than tissue biopsy, or even radiologic imaging [58]. These vesicles also have the ability to be exogenously altered and injected, therefore giving them pharmacotherapeutic use as well [26]. Exosomes show promise in their potential to provide specific insights about physiological functions as well as disease, and prostatic malignancies are no exception. For example, exosomes isolated from the plasma of patients with CRPC are significantly smaller in hydrodynamic size than those isolated from patients with localized prostate cancer [52]. Del Re et al. demonstrated that the detection of AR-V7 in exosomal RNA is predictive of resistance to hormone therapy in metastatic prostate cancer [14]. Significantly enough, emerging evidence suggests that fatty acid-binding protein 5 (FABP5) in extracellular vesicles is significantly associated with Gleason score in prostate cancer patients [53].

### 2.4. The Biomarker Value of Exosomes in Prostate Cancer

**Figure 2:**
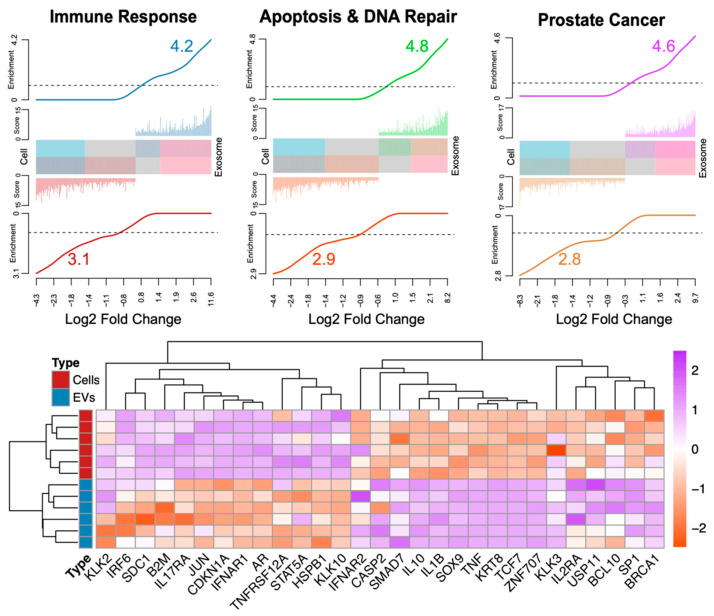
Barcode plot figure [68] showing significantly higher enrichments in exosomes compared to cells associated with immune response (n = 236 transcripts), apoptosis and DNA repair (n = 200), and prostate cancer (n = 262). The barcode plot shows the log2 fold change in the X-axis. The Y-axis is composed by score (-log10 false discovery rate or adjusted *p* values) and enrichment (gene set-weighted density estimation). The middle bar and colored histogram in either top or bottom of the bar reflect the number of differentially expressed genes (adjusted *p* values < 0.05). The bottom section of the figure shows the standardized expression profiles of genes associated to the pathways showed in the top section. The data in this figure are available through Dogra et al. [37], representing the comparison between patient tissues and serum-extracted exosomes.

Currently, the lineage of tumor-derived EVs remains poorly understood. This, in part, is due to the relatively small occurrence of tumor-derived EVs among all EVs that are accumulated from different cell types in biofluids. For instance, ultracentrifugation (UC), size-exclusion chromatography (SEC), and nanoparticle tracking analyses (NTA) have shown that human urine contains ~10–100 million EVs per mL, which are accumulated from all tissue/cell types and only a fraction originates from the tumor tissue of interest [59,60]. Nevertheless, exosomes are enriched with immune response, apoptosis, DNA repair, and prostate cancer gene signatures (Figure 2). It remains poorly understood whether the prostate-specific signatures exist in biofluid-derived EVs and if such biomarkers can be reproducibly studied for liquid biopsy. To address this, we employed a literature-curated prostatic gene set (Figure 2).

To understand whether the prostate-specific signatures are present in the circulating EVs, we curated a set of 22 widely studied and published markers, which are either prostate-specific or cancer-specific. For instance, PSA-encoding kallikreins (KLK2, KLK3, and KLK4), androgen receptor (AR), and NKX3-1 are exclusively expressed in prostate tissue [8]. Additionally, prostate tissue is largely comprised of specific markers expressed in epithelial (EPCAM, EGFR, KRT8), basal (BCAM), and stromal cells (CD44, CD105, CD29). Hence, combining all prostate tissue-specific markers may yield an overall prostate tissue lineage gene set, which can be used to label exosomes as being of prostatic origin. In contrast, markers expressed in the cells of endothelial (ICAM1, MCAM, Sele) and leucocyte origin, from markers expressed in the cells of hematopoietic lineage (CD45, CD16, CD41), are known to be absent in prostatic tissue and can be used as negative prostatic markers. Recently, many ncRNAs, such as MALAT1, NEAT1, MIR25, and LET-7, are found to be associated with prostate cancer [61,62]. Moreover, similar RNA lineage studies have previously been conducted for CTCs [63]. Taken together, a prostate-specific exosome signature will likely contain proteomic and/or genomic traces of kallikreins, AR, NKX3-1, PSA, and PSMA (Figure 2). Numerous strategies, such as immunoprecipitation and single-cell RNAseq, may be applied for disease diagnosis, monitoring, treatment response, and prognostication. With a thorough understanding of their function, EVs can be used as a minimally invasive and highly specific diagnostic and therapeutic tool for prostate cancer.

The identification and validation of novel biomarkers of cancer progression towards defining cancer type and subtype, stage, and optimized therapeutic response is an ongoing and challenging pursuit by investigative teams [64]. Specific molecules, such as cell-free DNA (cfDNA), CTCs, RNAs, cell-free proteins, and exosomes, are all of specific interest when it comes to the diagnostic utility of liquid biopsies [65]. cfDNA is present in over 70% of urine samples in patients with renal cell carcinoma (RCC), prostate cancer, and bladder cancer. The identification and analysis of cfDNA may be helpful, due to the fact that they contain tumor-specific genetic and epigenetic alterations. Studies have shown significant differences in cfDNA levels in cancerous and noncancerous patients, with the former having an average of 180 ng/mL of blood, and the latter having only 30 ng/mL [66]. In metastatic RCC, elevated cfDNA levels predict postoperative recurrence with 91% sensitivity and 100% specificity. In prostate cancer, cfDNA levels are significantly higher than in patients with BPH [65]. cfDNA integrity is also related to the presence of RCC, higher stage, and greater tumor size. Horning et al. demonstrated that in patients with biochemical recurrence following radical prostatectomy, the plasma cfDNA methylation of SRD5A2 and CYP11A1 was elevated, indicating that cfDNA may be useful in detecting prostate cancer recurrence [67].

CTCs are cells that are released into circulation from a tumor or site of metastasis. CellSearch is an FDA-approved system for the enrichment and detection of CTCs, and according to the platform, having five or more CTCs per 7.5 mL of blood corresponds to poor prognosis in patients with mCRPC [69]. One study found that CTCs were detectable in COPD patients without clinically detectable lung cancer one to four years before lung nodules were found on the CT scan [70]. CTCs have also been found to be elevated in RCC, and the levels are correlated to the stage and aggression of the disease [65]. Similar trends have been observed in colorectal cancer, bladder cancer, liver cancer, and esophageal cancer [20].

Circulating RNAs, including mRNAs, ncRNAs, and lncRNAs, have also been shown to change in cancer, but return to normal after surgery in cancers such as RCC and bladder cancer [65,71]. ncRNA serves as a promising potential biomarker, due to its ability to be detected from isolated exosomes [72]. In patients with CRPC, who also exhibit resistance to first-line taxane chemotherapy, especially those who are resistant to docetaxel, the serum levels of miR-21 are significantly elevated [73]. Another study showed that the analysis of miR-1290 and miR-375 can predict CRPC survival [74]. PCA3, an lncRNA, has already been approved by the FDA, for clinical use as a urine test, to decide whether or not repeat prostate biopsies should be completed [65]. Detecting the plasma levels of MALAT-1, another lncRNA, can help prevent between 30 and 46% of unnecessary prostate biopsies in men with slightly elevated PSA levels [75]. Circulating mRNAs provide another potential biomarker tool for prostate cancer, with AR-V7 levels serving as potential predictive markers in mCRPC, since higher levels have been linked to worse prognosis [76]. 

Cell-free proteins and peptides provide biomarker utility as well [77]. Commercially available protein marker tests for urologic malignancies include the prostate health index and 4KScore for prostate cancer, ImmunoCyt for urothelial carcinoma, and the Aura Tek FDP test for bladder cancer recurrence. The detection of CAV1 and CAV2 in urine can be used to distinguish between CRPC and non-CRPC, and 12 urinary peptides have been found to be able to distinguish between prostate cancer and benign conditions [65]. The presence of exosomes in all bodily fluids serves as the basis for their diagnostic significance in liquid biopsies. Exosomes can be isolated in several different ways, the most common of which is serial centrifugation. Vesicle size, as well as the presence of certain cell markers, may be used to ensure the presence of pure exosomes—these markers include CD9, CD63, and Alix, while markers such as calnexin should not be present in the ultrafiltrate [21]. Exosomes can be visualized using confocal or high-resolution microscopes. However, the techniques described to date describe isolating and tagging the exosomes with fluorescently tagged antibodies to proteins present inside the exosome or localized to their membrane. Such visualization can be employed to analyze specimens ex vivo or used to track the localization of exosomes after their uptake by recipient cells in vivo [78,79].

Exosomes have shown great promise in cancer diagnosis (Table 2), due to their high degree of stability and their high levels in cancer patients compared to people without cancer. Exosomes are highly enriched in RNAs compared to their cellular counterparts [80]. Their RNA cargo is associated with critical biological processes and molecular biomarkers that reflect disease states such as cancer [37,80]. More importantly, approximately 44% of the cargo RNA transcripts are composed of uncharacterized ncRNAs, representing an unexplored source of potentially medically relevant biomarkers [37]. Figure 2 reveals a summary of the gene enrichments for genes (between exosomes and cells) contained within the following: (1) immune response pathways (TNF gamma, IFN alpha, IL6-Jak-Stat3, complement, and IL2-Stat5), reflected in the higher exosome expression of genes such as IRF6, IFNAR1, TNFRSF12, and STAT5A; (2) apoptosis and DNA repair (NFK-β, KRAS, DNA damage response, mitotic spindle, and response to ROS), reflected by the higher exosome expression of genes such as HSPB1 and SDC1; and (3) prostate cancer (P53, MYC, Wnt, androgen/estrogen response, and angiogenesis), with higher exosome expression of the genes AR, KLK2, CDKN1A, KLK10, JUN, and B2M, compared to their cellular counterparts. These observations strongly suggest preferential exosomal packaging of highly informative critical RNA transcripts that report disease and may be used as non-invasive biomarkers.

The potential diagnostic utility of exosomes in prostate cancer was implicated by a series of studies. Nilsson et al. showed that tumor exosomes isolated from urine could be used to detect and amplify PCA3 and TMPRSS2:ERG mRNA, even with a very small amount of exosomal RNA [23]. One study found that survivin, an apoptosis inhibitor gene, was present at higher levels in exosomes isolated from the plasma of patients with prostate cancer than in those without. Increased survivin levels corresponded to higher Gleason scores, regardless of recurrence or BPH, making survivin a potential exosomal marker for early prostate cancer detection [54]. Using liquid biopsies potentially provides several benefits, including comfort, repeatability, less complexity in acquisition, and low cost, compared to tissue biopsies [81]. Thus, frequent samples can be taken to follow the progression of disease and therapeutic response, ultimately impacting patient survival outcomes.

### 2.5. Therapeutic Value of Exosomes in Prostate Cancer

The targeting value of exosomes is still under evolution, despite growing evidence recognizing its potential (Table 3). Significantly enough, their first attractive feature is that they are well tolerated and do not induce toxicity, even when injected repeatedly [26]. In therapy, as is the case with diagnostic testing, the protection of their contents from degradation, due to their membranes, serves as an effective tool for content delivery. Targeted exosome delivery has had success in reaching and changing protein expression in the central nervous system, as well as in breast cancer cells. Nontargeted exosome delivery may also be used, with the increased risk of affecting surrounding tissue [21].

In the bladder, urothelium cancerous cells have been found to be 50 times more likely to uptake exosomes than normal neighboring urothelium [82]. When exosomes containing PLK-1 siRNA are delivered to bladder cancerous cells, PLK-1 mRNA has been shown to subsequently decrease—demonstrating that exosomes as a method of therapy delivery can be beneficial [21]. Exosomes have also been shown to uptake and deliver pharmacological agents to cancerous tissue. This was shown with doxorubicin in breast cancer cells containing iRGD, where targeted exosome delivery in mouse models led to preferential drug concentration in tumor tissue, and tumor growth decreased more rapidly than when systemic doxorubicin was provided [83].

**Table 3:** Summary of evidence on potential value of exosomes in cancer therapeutics including prostate tumors.

Reference	Cancer Type	Therapeutic Application of Exosomes in Cancer
[83]	Breast	Targeted exosome delivery of doxorubicin to tumor tissue leads to more rapid tumor regression than systemic doxorubicin therapy
[82]	Bladder	Cancerous cells are 50 times more likely than neighboring urothelium to take up exosomes
[21]	Bladder	Exosome delivery of PLK-1 siRNA to cancer cells significantly reduces PLK-1 mRNA
[84]	Prostate	Knockdown of ACTN4 gene (highly expressed in exosomes of CRPC patients) diminishes invasion and proliferation of prostate cancer cells
[85]	Prostate	ASC-derived exosomal miR-145 promotes prostate cancer apoptosis via caspase-3/7 pathway
[86]	Prostate	Cancer cell-derived exosome delivery of paclitaxel increases drug cytotoxicity

The current treatment options for CRPC have limitations, which have created a need for new therapeutic angles [87]. Ishizuya et al. demonstrated that the knockdown of the gene encoding actinin-4, an exosomal protein that is selectively overexpressed in patients with CRPC or untreated metastatic prostate cancer, can successfully suppress prostate cancer cell growth and invasion [84]. Mechanistically, exosomal miR-145 from adipose-derived stromal cells, or ASCs, may also serve as a therapeutic target for prostate cancer, as it has been shown to reduce Bcl-xL activity and promote prostate cancer cell apoptosis via the caspase-3/7 pathway [85]. Of major translational significance is the evidence by Saari and colleagues demonstrating that the antitumor effect of paclitaxel taxane chemotherapy is enhanced when delivered by cancer cell-derived exosomes [86].

## 3. Conclusions and Future Directions

Prostate cancer serves as an ideal candidate for liquid biopsy, due to the anatomical location of the prostate gland, allowing for a great amount of shedding of exosomes into the urine. With the high burden that prostate cancer has on men throughout the world, it is imperative to focus on the pursuit of precise biomarkers detecting the emergence of advanced tumors, and ultimately impairing the lethal disease. Currently, there is an intense pursuit for highly sensitive and specific biomarkers for prostate cancer that can be isolated from exosomes. Once this occurs, additional progress can be made to streamline the diagnostic process and make it clinically accessible. The long-term goal of establishing the clinical use of exosomes in diagnostics and therapeutics is to reduce unnecessary biopsies, overdiagnosis, and overtreatment of prostate cancer, while increasing the ability to detect high-risk cancers that may have been missed with a PSA test. One must recognize that further work is needed in the development of new pharmacotherapeutic tools for prostate cancer—especially for CRPC. Exosomes provide significant value in improving the prognosis of localized and advanced prostate cancer, as well as predicting therapeutic resistance and tumor relapse in patients with the lethal disease.

## Figures and Tables

**Table 1:** Evidence linking exosomes to tumor progression and therapeutic resistance.

Reference	Cancer Type	Exosomal Correlation
[47]	Glioma	Exosomes transport EGFRvIII to cells that did not previously have it
[48]	Pancreatic	Cancer cell exosomes are able to instigate malignant cell transformation
[49]	Colon	Exosomes can mediate transfer of mutant KRAS to wild-type colon cells
[50]	Breast	Exosomal ncRNA can confer drug resistance in breast cancer
[51]	Prostate	Exosomes confer docetaxel resistance from cell to cell
[52]	Prostate	Plasma-derived exosomes derived from patients with CRPC are significantly smaller than those from patients with localized disease
[14]	Prostate	Detection of AR-V7 in exosomal RNA can predict resistance to hormone therapy in metastatic disease
[53]	Prostate	Higher exosomal FABP5 content is correlated with higher Gleason score prostate cancer
[54]	Prostate	Increased exosomal survivin levels correspond to higher Gleason scores

**Table 2:** Summary of the biomarker value of exosomes in cancer.

References	Biomarker Value of Exosomes in Cancer
[37]	44% of exosomal cargo transcripts are comprised of ncRNAs with potential biomarker utility
[37]	Higher exosomal gene expression of immune pathway genes such as IRF6, IFNAR1, TNFRSF12, and STAT5
[37]	Higher exosomal gene expression of apoptosis and DNA repair genes such as HSPB1 and SDC1
[37]	Higher exosomal gene expression of androgen-regulated genes such as AR, KLK2, CDKN1A, KLK10, JUN, and B2M
[23]	PCA3 and TMPRSS2:ERG mRNA can be isolated from urinary exosomes
[54]	Prostate cancer patient plasma contains higher levels of survivin

## Data Availability

The data in Figure 2 are available through Dogra et al. [37], representing the comparison between patient tissues and serum-extracted exosomes.

## Abbreviations

ALIX	Apoptosis-linked gene 2-interacting protein X
APC	Antigen-presenting cell
AR	Androgen receptor
AR-V7	Androgen receptor splice variant 7
ASC	Adipose-derived stromal cell
Bcl-xL	B-cell lymphoma-extra large
BPH	Benign prostatic hyperplasia
cfDNA	Cell-free DNA
CRPC	Castration-resistant prostate cancer
CTC	Circulating tumor cell
DNA	Deoxyribonucleic acid
DRE	Digital rectal exam
ESCRT	Endosomal sorting complexes required for transport
ESE	Early sorting endosome
EMT	Epithelial–mesenchymal transition
EV	Extracellular vesicle
FABP5	Fatty acid-binding protein 5
FasL	Fas ligand
HSP	Heat shock protein
ICAM-1	Intercellular adhesion molecule 1
ILV	Intraluminal vesicle
iRGD	Internal arginylglycylaspartic acid
KLK	Kallikrein
LSE	Late sorting endosome
mCRPC	Metastatic castration-resistant prostate cancer
MET	Mesenchymal–epithelial transition
mRNA	Messenger RNA
MHC-II	Major histocompatibility complex class II
MVB	Multivesicular body
ncRNA	Noncoding RNA
NTA	Nanoparticle tracking analysis
PCA3	Prostate cancer antigen 3
PCPT	Prostate cancer prevention trial
PSA	Prostate-specific antigen
PSMA	Prostate-specific membrane antigen
RCC	Renal cell carcinoma
RNA	Ribonucleic acid
SEC	Size-exclusion chromatography
siRNA	Small interfering RNA
TMPRSS2:ERG	Transmembrane protease serine 2:ETS (erythroblast transformation-specific)-related gene
UC	Ultracentrifugation
